# Possible Involvement of Foxp3^+^ Regulatory T Cells in the Development of Immune-Mediated Pancreatitis in MRL/Mp Mice Treated with Polyinosinic:Polycytidylic Acid

**DOI:** 10.1155/2013/367325

**Published:** 2013-05-27

**Authors:** Masanori Koyabu, Kazushige Uchida, Yutaku Sakaguchi, Norimasa Fukata, Takeo Kusuda, Hideaki Miyoshi, Katsunori Yoshida, Kimi Sumimoto, Toshiyuki Mitsuyama, Toshiro Fukui, Akiyoshi Nishio, Kazuichi Okazaki

**Affiliations:** The Third Department of Internal Medicine, Division of Gastroenterology and Hepatology, Kansai Medical University, 10-15 Fumizono, Moriguchi, Osaka 570-8507, Japan

## Abstract

*Objectives*. This study was conducted to clarify whether or not Tregs are involved in the development of immune-mediated pancreatitis in MRL/Mp mice as an AIP (autoimmune pancreatitis) model, in order to understand more clearly the pathogenic mechanism of AIP. *Methods*. We compared the immunohistochemical features of pancreatic forkhead box P3 (Foxp3) in the administration of poly I:C in MRL/Mp mice and two types of control mice (BALB/c and C57BL/6). As a contrast, we analyzed three mouse models of pancreatitis without autoimmune mechanism (Cerulein-, Ligation-, and Ligation + Cerulein-treated mice). After staining these specimens, we compared the ratios of Foxp3-positive cells to infiltrated mononuclear cells (Foxp3/Mono). *Results*. Our immunohistochemical study of Foxp3 revealed that the infiltration of Foxp3-positive cells increased in poly I:C-treated MRL/Mp mice. The histopathological score of pancreatitis showed no difference among poly I:C-treated MRL/Mp, Ligation-, and Ligation + Cerulein-treated mice; however, the Foxp3/Mono ratio in poly I:C-treated MRL/Mp mice was significantly increased compared with Ligation- and Ligation + Cerulein-treated mice. *Conclusions*. MRL/Mp mice treated with poly I:C showed early development of pancreatitis with abundant infiltration of Foxp3-positive cells. There may be a possibility that Tregs are involved in the development of pancreatitis in these mice.

## 1. Introduction

Recently, autoimmune pancreatitis (AIP) has been accepted worldwide as a unique, distinctive disease, in which histopathological findings show abundant infiltration of IgG4-positive plasma cells and fibrosis, known as lymphoplasmacytic sclerosing pancreatitis (LPSP), and clinical manifestations that respond dramatically to steroid therapy. Patients with AIP generally show the presence of several autoantibodies. Although a disease-specific antibody has not been identified at this moment, the antilactoferrin antibody (A-LF), anticarbonic anhydrase (A-CA), and antipancreatic secretory trypsin inhibitor (PSTI) autoantibodies have been frequently detected in Japanese patients with AIP [1–4]. Carbonic anhydrase (CA)-II, actoferrin (LF), and PSTI are distributed in the ductal cells of several exocrine organs, including the pancreas, the salivary gland, the biliary duct, and distal renal tubules. The high prevalence of these antibodies suggests that they are candidate target antigens in AIP. The precise mechanism underlying the onset of AIP and its progress is still unknown [5], although diagnosis and therapy have been investigated extensively with significant progress. However, it is difficult to prove whether autoimmune mechanisms are involved in the development of pancreatitis [6]. Therefore, establishing an animal model for AIP is critical. Several animal models have been reported: alymphoplasia (aly^−/−^) mice [7], MRL mice [8, 9], nTx-NFS/sld mice [10], and WBN/Kob rats [11]. Even though some spontaneous animal models partially resemble human AIP, there are many differences in terms of symptoms, gene functions, and pathological findings. It has been shown that the administration of polyinosinic-polycytidylic acid (poly I:C, a Toll-like receptor 3 ligand) accelerates immune-mediated pancreatitis in MRL/Mp mice, an autoimmune susceptible model of pancreatitis [12]. Immunohistochemical examination revealed predominant infiltration of CD4^+^ T cells and Mac-2^+^ activated macrophages in poly I:C-induced pancreatic lesions.

CD4^+^CD25^+^ regulatory T cells (Tregs) develop in the thymus and in the periphery and actively maintain immunological self-tolerance. Tregs are characterized by the expression of a specific transcription factor, forkhead box P3 (Foxp3), and play a key role in the autoimmune diseases. We previously reported how we developed AIP animal models by immunizing neonatally thymectomized (nTx) mice with CA-II and LF, and also by transferring immunized spleen cells to nude mice [13]. In this model, the neonatal thymectomization removes Tregs from the periphery [14], which induces a hyperimmune state. Therefore, we suggested that MHC class II-restricted CD4^+^ T cells react to CA-II or LF, which allows them to escape from negative selection in the thymus and depletion of Tregs in the periphery, thus having important roles in the development of AIP in nTx mice. Our previous data also showed that increased numbers of CD4^+^CD25^high^ Tregs may influence IgG4 production, and naïve Tregs may be involved with pathogenesis in AIP patients [15]. However, the relationship of Tregs in the peripheral blood and the pancreas still remains unclear. The purpose of this study is to clarify the difference in administration of poly I:C in three strains of mice (MRL/Mp, BALB/c, and C57BL/6), and in three mouse models of pancreatitis without autoimmune mechanism (Cerulein-, Ligation-, and Ligation + Cerulein-treated mice) by making a comparative study of the immunohistochemical features of the pancreas. These 3 mouse models can serve as models for acute, chronic, and severe pancreatitis [16].

## 2. Materials and Methods

### 2.1. Mice

Six-week-old female MRL/Mp mice were purchased from The Jackson Laboratory (Bar Harbor, ME, USA). Six-week-old female BALB/c and C57BL/6J mice were purchased from CLEA Japan, Inc. (Osaka, Japan). All mice were maintained in our animal center under specific pathogen-free conditions. All experiments were approved by the Animal Experimentation Committee, Kansai Medical University. Animals were randomly distributed into the different groups as indicated.

### 2.2. Mice Treatment

Poly I:C (Sigma Chemical Co, St Louis, MO, USA) was injected intraperitoneally at a dose of 5 mg/kg once every 3 days, starting when MRL/Mp mice were 6 weeks old and continuing until they were 10–20 weeks old [12]. Six-week-old female BALB/c and C57BL/6J mice were injected with Poly I:C until they were 18 weeks old. The controls received the same volume of carrier solution. Cerulein-treated mice, Ligation-treated mice, and Ligation + Cerulein-treated mice were treated as previously described [16]. Cerulein-treated mice were composed of mice intraperitoneally injected with cerulein (Sigma, St Louis, Mo), a cholecystokinin analogue, 7 times on day 0 (dosage, 50 mg/g every hour) to make an acute pancreatitis model. Ligation-treated mice were composed of mice whose pancreatic segment close to the spleen (pancreatic tail) was exposed through a midline incision under diethyl ether anesthesia. The pancreatic duct from this part was double ligated to make a chronic pancreatitis model. Ligation + Cerulein-treated mice were composed of B6 mice treated with the double ligature of the pancreatic duct plus the injection of cerulein on day 0 to make a severe pancreatitis model.

### 2.3. Histopathology

The pancreases were fixed in 3% phosphate-buffered formalin and embedded in paraffin. From the paraffin-embedded tissue blocks, 5 *μ*m sections were serially cut and stained with hematoxylin-eosin for routine histology assessment. A light microscope was used for histopathological evaluation of the pancreatic lesions. The severity of each lesion in at least one section of pancreas was scored on a 0–4 grade on the basis of the histopathological changes as previously described [8, 12]: briefly, (0) pancreas without mononuclear cell infiltration, indicating it is almost normal; (1) mononuclear cell aggregation and/or infiltration within the interstitium without any parenchymal destruction; (2) focal parenchymal destruction with mononuclear cell infiltration; (3) diffuse parenchymal destruction but with some parenchymal residue retained intact; (4) almost all pancreatic tissue, except pancreatic islets, destroyed or replaced with fibrosis or adipose tissue. The maximum score was used as the pancreatitis grade of each individual. To estimate the incidence of pancreatitis; mice with pancreatic lesions that scored > 2 were defined as positive.

### 2.4. Immunostaining of Foxp3

Formalin-fixed and paraffin-embedded specimens were prepared and used forimmunohistochemical studies. The Foxp3 immunostaining utilized the avidin-biotin complex (ABC) method with reagents provided by Vector Laboratories (Burlingame, CA, USA), using biotin-conjugated anti-mouse Foxp3 (eBioscience, San Diego, CA, USA). The deparaffinized sections were pretreated in an ethylenediaminetetraacetic acid buffer (pH 8.0) in a pressure-cooker at 100°C for 5 min. The sections were incubated with the first antibody at 4°C overnight, using 3,3′-Diaminobenzidine tetrahydrochloride (DAB) as the chromogen. The numbers of immunohistochemically identified Foxp3-positive cells and mononuclear cells contained within the pancreas selected in each specimen were counted under five different high power fields (hpf), and the ratios between Foxp3-positive cells and infiltrated mononuclear cells were calculated in each case.

### 2.5. Dual Fluorescent Immunostaining of Foxp3 and CD4

Half of the pancreases were embedded in OCT compound (Miles, Elkhart, IN, USA) and stored at −70°C before cutting. Biotin-conjugated anti-mouse Foxp3 (eBioscience, San Diego, CA, USA) was used for Foxp3 immunostaining. Sections were incubated with the first antibody at 4°C overnight. The sections were incubated for 10 min at room temperature with Cy3-conjugated streptavidin (Jackson ImmunoResearch Laboratories, West Grove, PA, USA). Then, the sections were incubated with fluorescein isothiocyanate (FITC)-conjugated anti-mouse CD4 (eBioscience) for one hour. Nuclei were stained with 4,6-diamidino-2-phenylindole (DAPI) (Vector Laboratories, Burlingame, CA, USA). The stained samples were analyzed using an optical microscope (BX50, Olympus, Tokyo, Japan).

### 2.6. Statistical Analysis

For all studies, data are expressed as mean ± SEM; differences were analyzed using the nonparametric Mann-Whitney rank test and Fisher's exact test, where *P* values less than 0.05 were considered significant.

## 3. Results

### 3.1. Poly I:C Induced Early Development of Immune-Mediated Pancreatitis in MRL/Mp Mice

**Figure 1:**
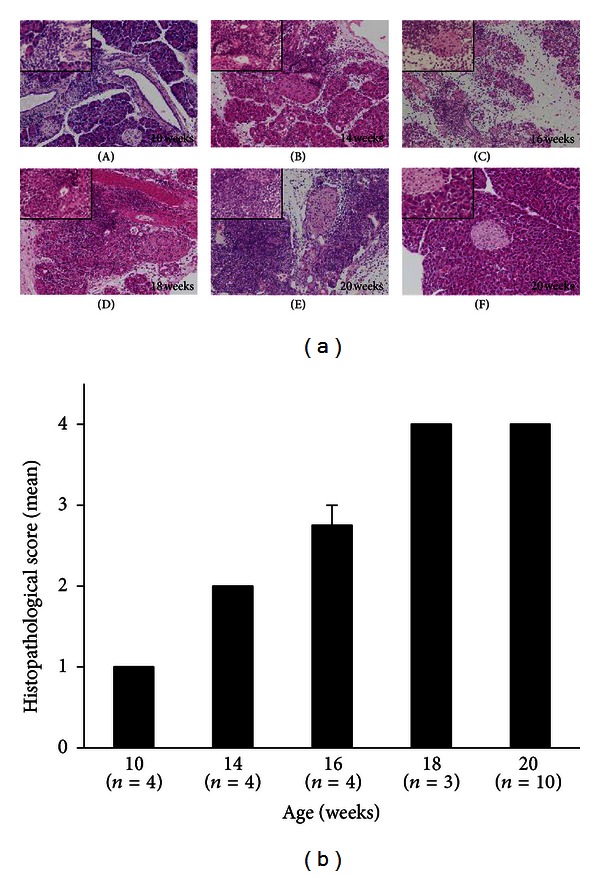
Representative H&E stained histological sections of the pancreases (a) and histopathological scores of the pancreases (b) in poly I:C-treated MRL/Mp mice in the 5 different age groups. The group of 10-week-old mice showed mononuclear cell aggregation and infiltration within the interstitium, and no pancreatic parenchymal destruction (grade 1). The group of 14-week-old mice showed focal parenchymal destruction with mononuclear cell infiltration in the pancreases (grade 2). The group of 16-week-old mice showed diffuse parenchymal destruction, but some parenchymal residue was retained intact in the pancreases (grade 3). The groups of 18- and 20-week-old mice showed that almost all pancreatic tissue, except for the pancreatic islets, was destroyed or replaced with fibrosis or adipose tissue (grade 4). The control mice were no significant change (grade 0). Poly I:C-treated MRL/Mp mice (A)–(E), controls (F). ×100 magnification (large panel), ×400(inset). Data are expressed as mean ± SEM.

The histopathology of the pancreas was observed in poly I:C-treated MRL/Mp mice at the age of 10–20 weeks (Figure 1). In the group of 10-week-old mice, most pancreatic tissue showed mononuclear cell aggregation and infiltration within the interstitium without any parenchymal destruction (grade 1). The majority of 14-week-old mice showed focal parenchymal destruction with mononuclear cell infiltration (grade 2). The majority of 16-week-old mice showed diffuse parenchymal destruction but some intact parenchymal residue was retained (grade 3). The majority of 18- and 20-week-old mice showed almost all pancreatic tissue destroyed or replaced with fibrosis or adipose tissue, except for pancreatic islets (grade 4).

### 3.2. Foxp3-Positive Cells Increased in MRL/Mp Mice

**Figure 2:**
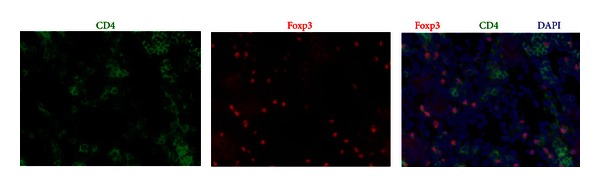
Detection of Foxp3-positive cells in the pancreas. Single immunostaining of CD4 (green; FITC) and Foxp3 (red; Cy3) in poly I:C-treated MRL/Mp mice at the age of 18 weeks. Nuclei were stained by DAPI (blue nuclear stain). The merged images demonstrated many mononuclear cells double positive for CD4 and Foxp3 in the pancreas. The most Foxp3-positive cells appeared in the CD4-positive cells (×400).

**Figure 3:**
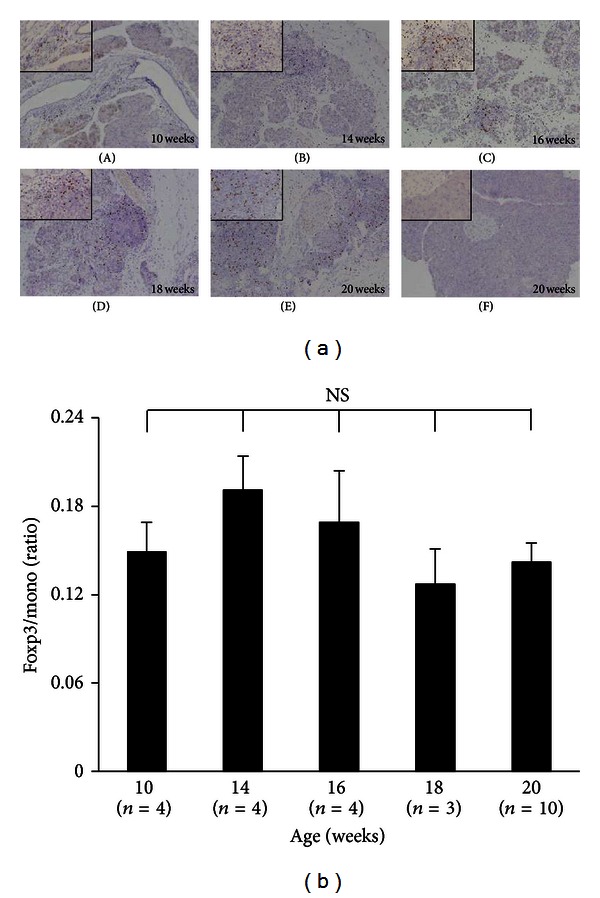
Representative immunostaining of Foxp3 in the pancreases (a) and the ratios of Foxp3/mono (Foxp3-positive cells/infiltrated mononuclear cells) in the pancreases (b) in poly I:C-treated MRL/Mp mice in the 5 different age groups. The abundant infiltration of Foxp3-positive cells was found among the lymphoid infiltrates in the pancreases. There were no differences in Foxp3/mono ratios among poly I:C-treated MRL/Mp mice at the ages of 10, 14, 16, 18, and 20 weeks. Poly I:C-treated MRL/Mp mice (A)–(E), controls (F). ×100 magnification (large panel), ×400(inset). Data are expressed as mean ± SEM; NS, not significant.

Foxp3-positive cells were examined by double immunostaining with CD4 and Foxp3, using specimens from the pancreases of MRL/Mp mice at the age of 18 weeks (Figure 2). Nuclei were stained with DAPI. The merged images demonstrated many mononuclear cells to be double positive for CD4 and Foxp3 in the pancreases. The majority of Foxp3-positive cells appeared in the CD4-positive cells. Foxp3 immunostaining and Foxp3/mono (Foxp3-positive cells/infiltrated mononuclear cells) ratios in the pancreas were observed in poly I:C-treated MRL/Mp mice in the 5 different age groups (Figure 3). Abundant infiltration of Foxp3-positive cells was found among the lymphoid infiltrates in the pancreases of poly I:C-treated MRL/Mp mice at the age of 10–20 weeks. There were no differences in the Foxp3/mono ratios among poly I:C-treated MRL/Mp mice at the ages of 10 (0.149 ± 0.02), 14 (0.191 ± 0.023), 16 (0.169 ± 0.035), 18 (0.127 ± 0.024), and 20 (0.142 ± 0.013) weeks.

### 3.3. Development of Immune-Mediated Pancreatitis Induced by Poly I:C and Infiltration of Tregs Depend on Mouse Strains

**Figure 4:**
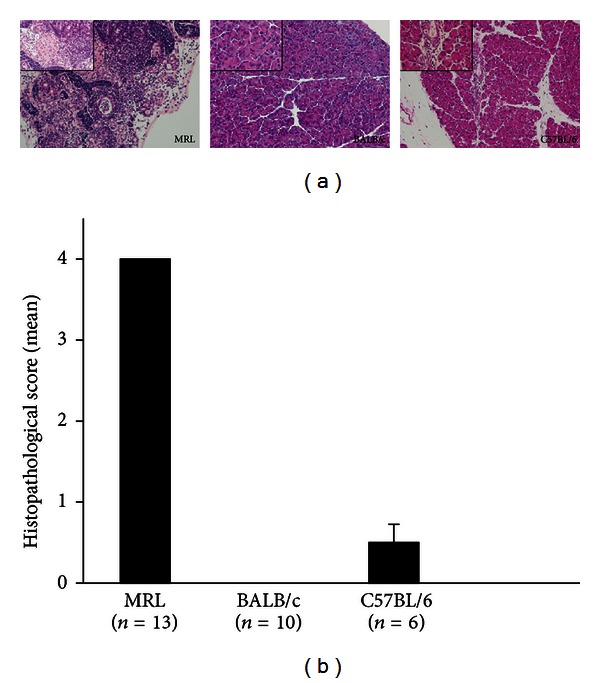
Representative H&E stained histological pancreatic sections of the pancreases (a) and histopathological pancreas scores (b) in poly I:C-treated MRL/Mp mice at the age of 18–20 weeks, poly I:C-treated BALB/c mice at the age of 18 weeks, and poly I:C-treated C57BL/6 mice at the age of 18 weeks. In MRL/Mp mice, representative pancreatic tissue showed almost all pancreatic tissue, except pancreatic islets, destroyed or replaced with fibrosis or adipose tissue (grade 4). In BALB/c mice, there was no significant change (grade 0). In C57BL/6 mice, pancreatic tissue showed mononuclear cell aggregation and infiltration within the interstitium without any parenchymal destruction (grade 1). ×100 magnification (large panel), ×400(inset). Data are expressed as mean ± SEM.

**Figure 5:**
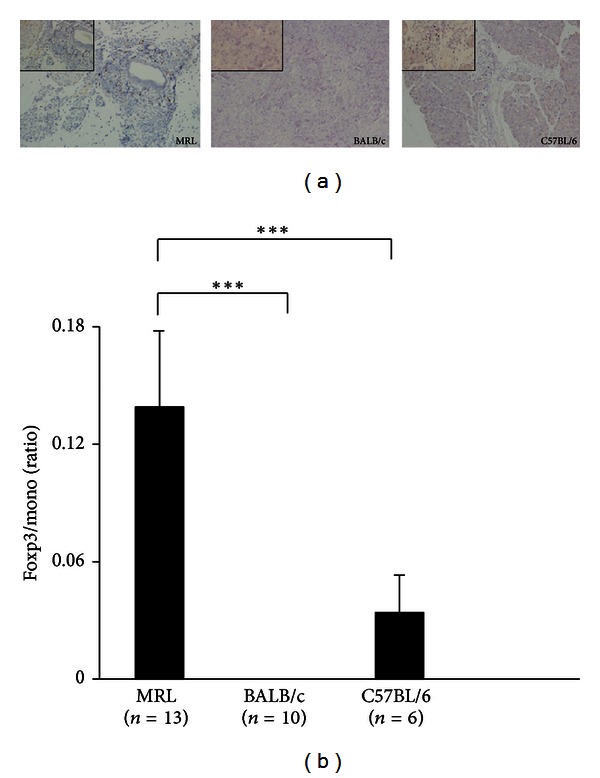
Representative immunostaining of Foxp3 in the pancreases (a) and the ratios of Foxp3/mono (Foxp3-positive cells/infiltrated mononuclear cells) in the pancreases (b) of poly I:C-treated MRL/Mp mice at the age of 18–20 weeks, poly I:C-treated BALB/c mice at the age of 18 weeks, and poly I:C-treated C57BL/6 mice at the age of 18 weeks. In MRL/Mp and C57BL/6 mice, abundant infiltration of Foxp3-positive cells was found among the lymphoid infiltrates in the pancreas but not in BALB/c mice. The Foxp3/mono ratios in MRL/Mp mice were significantly increased compared with BALB/c and C57BL/6 mice. ×100 magnification (large panel), ×400(inset). Data are expressed as mean ± SEM; ***P < 0.001.

The histopathology of the pancreas was observed in poly I:C-treated MRL/Mp mice at the age of 18–20 weeks, poly I:C-treated BALB/c mice at the age of 18 weeks, and poly I:C-treated C57BL/6 mice at the age of 18 weeks (Figure 4). In MRL/Mp mice, representative pancreatic tissue showed almost all pancreatic tissue, except pancreatic islets, destroyed or replaced with fibrosis or adipose tissue. In BALB/c mice, there was no significant change. C57BL/6 mice showed mononuclear cell aggregation and infiltration within the interstitium without any parenchymal destruction. The histopathological scores of the pancreases in MRL/Mp, BALB/c, and C57BL/6 mice were 4 ± 0, 0 ± 0, and 0.5 ± 0.224, respectively (Figure 4(b)). Foxp3 immunostaining and ratios of Foxp3/mono (Foxp3-positive cells/infiltrated mononuclear cells) in the pancreas were observed in poly I:C-treated MRL/Mp mice at the age of 18–20 weeks, poly I:C-treated BALB/c mice at the age of 18 weeks, and poly I:C-treated C57BL/6 mice at the age of 18 weeks (Figure 5). Abundant infiltration of Foxp3-positive cells was found among the lymphoid infiltrates in the pancreases of MRL/Mp and C57BL/6 mice, but not in BALB/c mice. The Foxp3/mono ratios in MRL/Mp mice (0.139 ± 0.039) were significantly increased compared with BALB/c (0 ± 0; *P* < 0.0001) and C57BL/6 mice (0.034 ± 0.019; *P* < 0.0001) (Figure 5(b)).

### 3.4. Tregs Infiltration Increased in Poly I:C Induced Immune-Mediated Pancreatitis in MRL/Mp Mice

**Figure 6:**
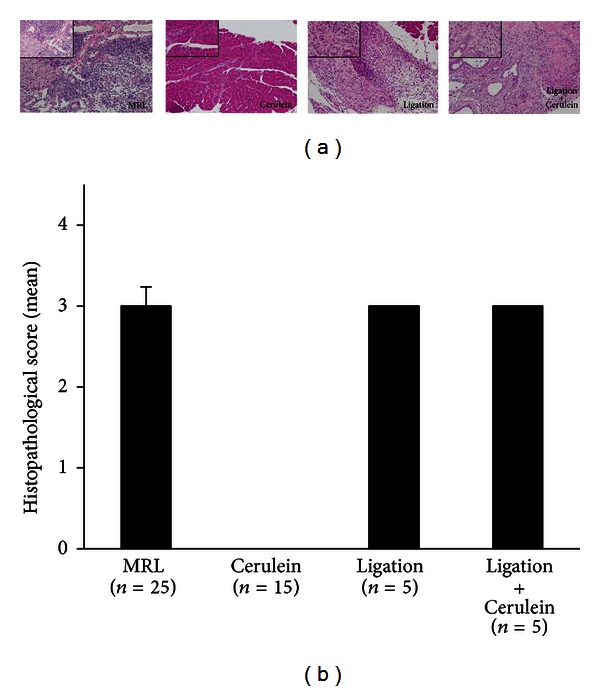
Representative H&E stained histological sections of the pancreases (a) and histopathological score of the pancreases (b) in poly I:C-treated MRL/Mp mice at the age of 10–20 weeks, along with Cerulein-, Ligation-, and Ligation + Cerulein-treated mice. In MRL/Mp, Ligation-, and Ligation + Cerulein-treated mice, representative pancreatic tissues showed diffuse parenchymal destruction, but with some parenchymal residue retained intact (grade 3). In Cerulein-treated mice, only slight edema was detected in the interstitium (grade 0). ×100 magnification (large panel), ×400(inset). Data are expressed as mean ± SEM.

**Figure 7:**
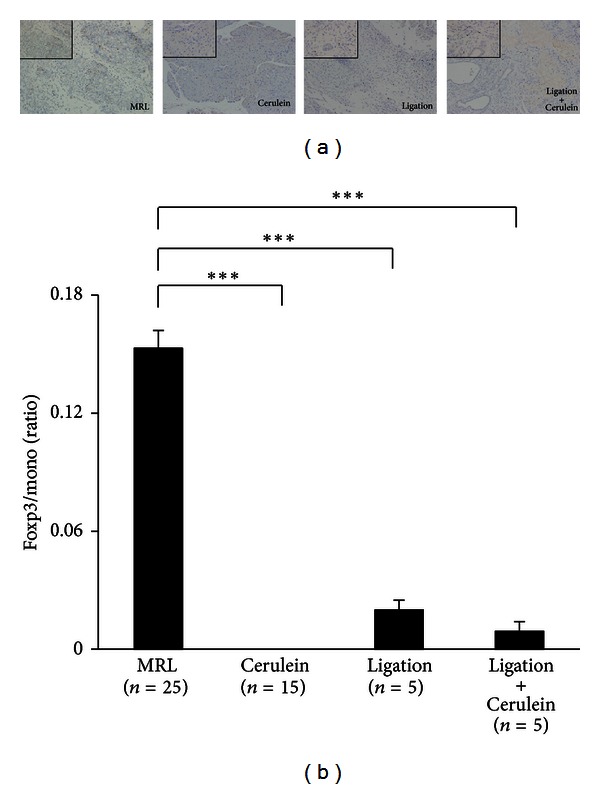
Representative immunostaining of Foxp3 in the pancreases (a) and the ratios of Foxp3/mono (Foxp3-positive cells/infiltrated mononuclear cells) in the pancreases (b) of poly I:C-treated MRL/Mp mice at the age of 10–20 weeks and Cerulein-, Ligation-, and Ligation + Cerulein-treated mice at the same age. Abundant infiltration of Foxp3-positive cells was found among the lymphoid infiltrates in MRL/Mp mice pancreases, but not in Cerulein-treated mice. In Ligation- and Ligation + Cerulein-treated mice, Foxp3 immunostaining of the pancreas showed infiltration of few Foxp3-positive cells. The Foxp3/mono ratios in MRL/Mp mice were significantly increased compared with Cerulein-, Ligation-, and Ligation + Cerulein-treated mice. ×100 magnification (large panel), ×400(inset). Data are expressed as mean ± SEM; ***P < 0.001.

The histopathology of the pancreas was observed in poly I:C-treated MRL/Mp mice at the age of 10–20 weeks as well as Cerulein-, Ligation-, and Ligation + Cerulein-treated mice at the same age. In MRL/Mp mice, Ligation-, and Ligation + Cerulein-treated mice, representative pancreatic tissues showed diffuse parenchymal destruction, but some parenchymal residue was retained intact. In Cerulein-treated mice, only slight interstitium edema was detected. The histopathological scores of the pancreases in MRL/Mp, Cerulein-, Ligation-, and Ligation + Cerulein-treated mice were 3 ± 0.238, 0 ± 0, 3 ± 0, and 3 ± 0, respectively (Figure 6(b)). Foxp3 immunostaining and Foxp3/mono ratios in the pancreas were observed in poly I:C-treated MRL/Mp mice at the age of 10–20 weeks, and also Cerulein-, Ligation-, and Ligation + Cerulein-treated mice (Figure 7). Abundant infiltration of Foxp3-positive cells was found among the lymphoid infiltrates in the pancreases of MRL/Mp mice, but not in Cerulein-treated mice. In Ligation- and Ligation + Cerulein-treated mice, pancreas Foxp3-immunostaining showed infiltration of few Foxp3-positive cells. The Foxp3/mono ratio in MRL/Mp mice (0.153 ± 0.009) was significantly increased compared with Cerulein-(0 ± 0; *P* < 0.0001), Ligation-(0.02 ± 0.005; *P* < 0.0001), and Ligation + Cerulein-treated mice (0.009 ± 0.005; *P* < 0.0001) (Figure 7(b)).

## 4. Discussion

This study was conducted to clarify whether Tregs are involved in the development of pancreatitis in MRL/Mp mice, in order to understand more clearly the pathogenic mechanism of AIP. Sarles et al. [17] observed the first case of pancreatitis with hypergammaglobulinemia. In 1995, Yoshida et al. [18] first proposed the concept of AIP, in which patients show a diffusely enlarged pancreas, a narrowing pancreatogram, increased serum IgG, the presence of autoantibodies, fibrotic changes with lymphocytic infiltration, and the efficacy of steroid treatment. Since that time, many AIP cases have been reported by Japanese investigators, and AIP has been accepted as a new clinical entity [1, 19, 20].

We used the MRL/Mp mice in this study as AIP model mice. An MRL/Mp mouse spontaneously develops severe autoimmune diseases such as glomerulonephritis, arteritis, sialoadenitis, and arthritis, associated with autoantibody production and T-cell dysfunction early in life [21–24]. MRL/Mp mice not bearing the *lpr* gene (MRL/+) also develop these diseases, but at a much later stage in life, and with reduced incidence and severity [22, 23], suggesting that the MRL strains have an autoimmune disease-like genetic background [24, 25]. We previously reported that serum IgG levels were increased in poly I:C-treated MRL/Mp mice compared with the control mice and CD4^+^ T cells infiltrated at the site of inflammation in poly I:C-treated MRL/Mp mice pancreases [24]. The pancreatitis accelerated by poly I:C in MRL/Mp mice was characterized by inflammatory cell infiltrates with acinar cell destruction and replacement by fibrous tissue and was selective for the exocrine pancreas, since it did not involve pancreatic islet cells. 

In this study, we administered Poly I:C to three strains of mice (MRL/Mp, BALB/c, and C57BL/6). In poly I:C-treated MRL/Mp mice, the pancreatic tissue showed diffuse parenchymal destruction, but some intact parenchymal residue retention. The severity of pancreatitis gradually increased in these mice. Poly I:C-treated BALB/c mice showed no significant pancreatic lesions. In poly I:C-treated C57BL/6 mice, the pancreatic tissue showed mononuclear cell aggregation and infiltration within the interstitium without any parenchymal destruction. Thus, the severity of the pancreatitis differed, depending on the strain of the mice. 

For the purpose of comparative studies, we also prepared three mouse models of pancreatitis without autoimmune mechanism (Cerulein-, Ligation-, and Ligation + Cerulein-treated mice). These three mouse models could serve as human models for acute, chronic, and severe pancreatitis [16]. In Ligation- and Ligation + Cerulein-treated mice, the pancreatic tissue showed diffuse parenchymal destruction but some intact parenchymal residue retention. In Cerulein-treated mice, only slight edema in the interstitium was detected, without infiltration of mononuclear cells.

In this study, we examined local infiltration of Tregs in the pancreas, because Tregs are reported to be involved in the development of the various autoimmune diseases. Tregs were initially found to efficiently control inflammation mediated by autoimmune diseases. Subsequently, in addition to autoimmune diseases, Tregs were implicated in controlling inflammation mediated by infectious pathogens and allergens [26–30]. As Tregs reside in lymphoid organs, early studies suggested that Tregs predominantly inhibit naive T-cell priming in the draining lymph nodes. Further studies show that Tregs infiltrate and move into inflammatory sites in various inflammatory diseases and suppress effector T-cell function. Tregs, consistent with their central role in the preservation of tissue integrity, can accumulate at inflammation or infection sites [31, 32]. Indeed, there is agreement among many investigators of various diseases that there is increased recruitment of CD4^+^CD25^high^ Tregs at inflammatory sites [33–37]. There are various subsets in Treg. CD4^+^CD25^+^ Tregs were important in the prevention of autoimmune diseases and were found to express high levels of Foxp3 [38–40]. Our immunohistochemical study of Foxp3 indeed showed an abundant infiltration of Foxp3-positive cells in poly I:C-treated MRL/Mp mice and few Foxp3-positive cells in poly I:C-treated C57BL/6 mice, but none in poly I:C-treated BALB/c mice. Foxp3 immunostaining of the pancreas showed an abundant infiltration of Foxp3-positive cells in 10-week-old MRL/Mp mice at the early stage of the pancreatitis. Therefore, it was thought that the ratio of Foxp3/mono was upregulated from the beginning in poly I:C-treated MRL/Mp mice. There was no difference in the Foxp3/mono ratio among poly I:C-treated MRL/Mp mice at the age of 10–20 weeks. The histopathological score of pancreatitis showed no difference among poly I:C-treated MRL/Mp, Ligation-, and Ligation + Cerulein-treated mice; however the Foxp3/mono ratio in poly I:C-treated MRL/Mp mice was significantly increased compared with Ligation- and Ligation + Cerulein-treated mice. It was thought that these Tregs were possibly involved in the development of immune-mediated pancreatitis in MRL/Mp mice. On the other hand, there was a possibility that the infiltration of Tregs in Ligation- and Ligation + Cerulein-treated mice was caused due to inflammation without autoimmune mechanisms. In this study, pancreatitis with abundant infiltration of Foxp3-positive cells developed in poly I:C-treated MRL/Mp mice therefore it may be possible that Tregs were related to the development of pancreatitis in poly I:C-treated MRL/Mp mice and that the function of infiltrated Tregs was different in MRL/Mp and C57BL/6 mice. These findings are similar to those humans [41]. On the other hand, recent studies have shown that CD4^+^CD25^+^ regulatory T cells in MRL/Mp mice display almost normal functions, with syngeneic CD4^+^CD25^−^ T cells showing significantly reduced sensitivity to suppression [42]. Further studies are necessary to clarify the function of Tregs in poly I:C-treated MRL/Mp mice. 

In conclusion, poly I:C-treated MRL/Mp mice showed possible involvement of Foxp3^+^ Tregs in the development of immune-mediated pancreatitis. Therefore, Tregs may possibly be related to the development of pancreatitis in these mice.
